# Epidemiology and pathophysiology of vascular thrombosis in acclimatized lowlanders at high altitude: A prospective longitudinal study

**DOI:** 10.1016/j.lansea.2022.05.005

**Published:** 2022-06-09

**Authors:** Velu Nair, Surinderpal Singh, Mohammad Zahid Ashraf, Uday Yanamandra, Vivek Sharma, Amit Prabhakar, Rehan Ahmad, Tathagata Chatterjee, Vineet Behera, Vivek Guleria, Seema Patrikar, Shivi Gupta, Madan Gopal Vishnoi, Rigvardhan, Kiran Kalshetty, Prafull Sharma, Nitin Bajaj, Thyelnai D. Khaling, Tanaji Sitaram Wankhede, Srinivasa Bhattachar, Rajat Datta, Late Prosenjit Ganguli

**Affiliations:** a Department of the Internal Medicine, Armed Forces Medical College, Pune, Maharashtra, India; b Director General Medical Services (Army), India; c Comprehensive Blood & Cancer Center (CBCC), Gandhinagar, Gujarat, India; d Department of Physiology, Armed Forces Medical College, Pune, Maharashtra, India; e Department of Physiology, Army College of Medical Sciences, New Delhi, India; f Defense Institute of Physiology & Allied Science (DIPAS), New Delhi, India; g Department of Biotechnology, Jamia Millia Islamia, New Delhi, India; h 153 General Hospital, Leh, India; i Department of Imaging & Radiodiagnosis, Armed Forces Medical College, Pune, Maharashtra, India; j Department of Radiology, Bharati Vidyapeeth Hospital & Medical College, Pune, Maharashtra, India; k Cardiovascular Research Institute (CVRI), University of California, San Francisco, USA; l Department of Clinical Haematology and Centre for Stem Cell Therapy and Research, Army Hospital (Research and Referral), New Delhi, India; m Luchkee Health Pvt Ltd Vasant Kunj, New Delhi, India; n Department of Laboratory Sciences and Molecular Medicine, Army Hospital (Research & Referral), New Delhi, India; o Department of Haematology and Stem Cell Transplant, ESIC Med College and Hospital, Faridabad, Haryana, India; p Department of Internal Medicine, INHS Asvini, Colaba, Mumbai, India; q Department of Cardiology, Army Hospital (Research & Referral), New Delhi, India; r Department of Community Medicine, Armed Forces Medical College, Pune, Maharashtra, India; s 403 Field Hospital, C/o 56 APO, India; t Indian Field Hospital, UN Mission, Malakal 71111, South Sudan; u Department of Nuclear Medicine, Command Hospital (Eastern Command), Kolkata, India; v Regimental Medical Officer, 20 Grenadiers c/o 56 APO, India; w Department of Anaesthesiology, 305 Field Hospital, C/o 99 APO, India; x Department of Cardiology, Military Hospital, Jalandhar, Punjab, India; y Department of Internal Medicine, Command Hospital (Western Command), Chandimandir, Haryana, India; z Department of Cardiology, Army Institute of Cardiothoracic Sciences, Pune, India; aa Department of Physiology, Institute of Aerospace Medicine, Bangalore, Karnataka, India; bb Department of Sports Medicine, Armed Forces Medical College, Pune, Maharashtra, India; cc High Altitude Medical Research Centre, Leh, Ladakh, India; dd Director General Armed Forces Medical Services, New Delhi, India; ee Department of Pathology, Command Hospital (Eastern Command), Kolkata, India

**Keywords:** Thrombosis, High altitude, Inflammation, Endothelial dysfunction, Fibrinolysis

## Abstract

**Background:**

Previous literature suggests that thrombosis is more common in lowlanders sojourning at high altitude (HA) compared to near-sea-level. Though the pathophysiology is partly understood, little is known of its epidemiology. To elucidate this, an observational prospective longitudinal study was conducted in healthy soldiers sojourning for months at HA.

**Methods:**

A total of 960 healthy male subjects were screened in the plains, of which 750 ascended, to altitudes above 15,000ft (4,472m). Clinical examination, haemogram, coagulogram, markers of inflammation and endothelial dysfunction, were studied at three time points during ascent and descent. The diagnosis of thrombosis was confirmed radiologically in all cases where a thrombotic event was suspected clinically. Subjects developing thrombosis at HA were labelled as Index Cases (ICs) and compared to a nested cohort of the healthy subjects (comparison group,(CG)) matched for altitude of stay.

**Findings:**

Twelve and three subjects, developed venous (incidence: 5,926/10^5^ person-years) and arterial (incidence: 1,482/10^5^ person-years) thrombosis at HA, respectively. The ICs had enhanced coagulation (FVIIa: p<0.001; FXa: p<0.001) and decreased levels of natural anticoagulants (thrombomodulin, p=0.016; tissue factor pathway inhibitor [TFPI]: p<0.001) and a trend to dampened fibrinolysis (tissue plasminogen activator tPA; p=0.078) compared to CG. ICs also exhibited statistically significant increase in the levels of endothelial dysfunction and inflammation markers (vascular cell adhesion molecule-1[VCAM-1], intercellular adhesion molecule-1 [ICAM-1], vascular endothelial growth factor receptor 3 [VEGFR-3], P-Selectin, CD40 ligand, soluble C-reactive protein and myeloperoxidase: p<0.001).

**Interpretation:**

The incidence of thrombosis in healthy subjects at HA was higher than that reported in literature at near sea-level. This was associated with inflammation, endothelial dysfunction, a prothrombotic state and dampened fibrinolysis.

**Funding:**

Research grants from the Armed Forces Medical Research Committee, Office of the Director General of Armed Forces Medical Services (DGAFMS) & Defence Research and Development Organization (DRDO), Ministry of Defence, India.

**Research in context**

**Evidence before this study**

The primary objective of this study, conducted from June 2012 to June 2014, was to determine the epidemiology, especially incidence, of thrombotic events in acclimatized healthy lowland soldiers, through the course of a two-year duty tenure at high altitude (HA)/extreme high altitude (EHA), along with the pathophysiological correlates at different time-points. We sought all published literature, systemic-analysis, meta-analysis and reviews available online up to 31 December 2010, in Pubmed, Google Scholar and Ovidsp, using the search terms high altitude, epidemiology, incidence, thrombosis, embolism, venous thromboembolism, deep vein thrombosis, cerebral/cortical vein thrombosis, stroke, infarction, peripheral arterial thrombosis, clotting, coagulation, fibrinolysis, platelet(s) and endothelium.

All literature on thrombotic events (venous/arterial) at HA/EHA in previously healthy lowland sojourners was reviewed. Most publications on the topic were retrospective case reports/series. These reports, suggested that thrombotic events might be commoner at HA but conspicuously did not report the incidence, except one (Kumar S. High altitude induced deep venous thrombosis: A study of 28 cases. Indian J of Surg 2006;68(2):84-88). This calculation was, however, based on hospital admissions of DVT of the calf veins only, using the “dependent” population number as the denominator. No prospective, population based epidemiological study could be found. This was, also, emphasized by Grover & Bartsch and vanVeen & Makris who bemoaned the absence of the knowledge of “the true incidence of thrombosis at high altitude” and “the absence of well-controlled epidemiological studies” on the topic (Grover RF, Bartsch P. Blood. In: Hornbien TF, Schoene RB, eds. High Altitude: An Exploration of Human Adaptation. New York: Marcel Dekker, 2001: 493-52, JJ vanVeen, M Makris. Altitude and Coagulation Activation: Does going high provoke thrombosis? Acta Haematol 2008;119:156-157).

Review of literature on the pathophysiology of thrombosis at HA/EHA showed that alterations of coagulation-fibrinolysis are reported in the setting of high-altitude illnesses such as AMS, HAPE, HACE and HAPH. However, in healthy subjects such changes were not reported either in the short or long-term. (Andrew M, O'Brodovich H, Sutton J. Operation Everest II: coagulation system during prolonged decompression to 282 torr. J Appl Physiol 1987; 63:1262–1267, Roeggla G, Roeggla M, Binder M, Wagner A. Does altitude or exercise induce fibrin degradation in mountaineers? J R Soc Med 1995; 889:239).

Investigations of cases of thrombosis at HA/EHA presented a limited repertoire of laboratory markers of the coagulation-fibrinolysis system, usually, haemoglobin/haematocrit, prothrombin and activated partial thromboplastin time, whereas more detailed studies of coagulation-fibrinolysis, endothelial/platelet activation and inflammation were done in subjects who did not develop thrombotic ailments. No study could be found where the entire sequence of chronic hypoxia-inflammation-endothelial activation-coagulation activation–anticoagulant &/or fibrinolysis inhibition had been studied in detail at HA or in subjects with thrombotic events at HA. Hence, there was an unmet need to study the epidemiology and pathophysiology of thrombotic events in soldiers stationed for prolonged periods at HA/EHA in the course of routine duty. This assumed greater importance as numerous case reports have highlighted the morbidity and mortality resulting from such thrombotic episodes. This study was precisely designed to address this need to **elucidate** the epidemiology and pathophysiology of thrombosis in healthy subjects at HA/EHA.

**Added value of this study**

We believe that this prospective observational study in healthy subjects presents the most robust estimate to date, of the incidence of thrombosis at high altitude. The reported incidence of venous and arterial thrombosis in a healthy population at HA/EHA is far in excess of that at near-sea-level; the latter including thrombosis in the general population, both the healthy and the sick. Hence, the real incidence in the general population at HA/EHA might be even higher.

To the best of our knowledge, this is the only report to date that provides a comprehensive study of the chronic hypoxia-inflammation-endothelial/platelet activation-coagulation-fibrinolysis axis in cases of thrombosis and matched healthy comparison group at HA/EHA. The findings suggest a clear relationship between ambient conditions (hypoxia at >15000ft) and hypercoagulation with dampened fibrinolysis, putatively linked by endothelial/platelet activation and inflammation, resulting in symptomatic thrombosis. SNP analysis of genetic variations commonly associated with thrombosis in cases and comparison group rounds off the study to give us a comprehensive view of the pathophysiological correlates of thrombosis at HA.

**Implications of all the available evidence**

The sum of all available evidence, including that generated in this study, suggests a hypercoagulable state in sojourners at HA/EHA and also a much greater incidence of thrombosis, compared to near-sea-level. This knowledge will help healthcare providers to develop preventive screening strategies for those ascending to HA/EHA. This provides a platform to launch further studies to identify high-risk subjects for thrombosis and any intervention to prevent the same. Further, knowledge of the molecular correlates at HA, will enhance understanding of the mechanisms of thrombosis not only at HA but also at near-sea-level.

Alt-text: Unlabelled box

## Introduction

Thrombosis, both arterial and venous, is a major cause of mortality and morbidity across the world.^1^ Venous thromboembolism (VTE), with a reported annual incidence of 0.7-2.69 per thousand, has a high recurrence rate, negative impact on survival, and incurs high healthcare costs.^2^ Arterial thrombosis constituting ischemic heart disease and stroke is responsible for one in four deaths worldwide.^1^, ^2^, ^3^ Some reports suggest that the occurrence of thrombosis is more at high altitudes (HA) than near sea level.^4^, ^5^, ^6^, ^7^, ^8^, ^9^ However, these are retrospective case reports/series which have not estimated the incidence of thrombosis at HA. The only study reporting the incidence of deep vein thrombosis (DVT) at HA to be 0.7 per 1000, was by Kumar but did not include cases of arterial and/or venous thrombosis at sites other than the calf veins.^10^ Reviewing the topic, Grover and Bartsch, as well as van Veen & Makris have bemoaned our ignorance of the true incidence and epidemiology of thrombosis at HA.^11^^,^^12^

We hypothesised that the incidence of thrombosis in healthy male adults at HA would be greater than reported in the general population at sea level. This may be attributed to the stasis of blood flow, increased viscosity, and a procoagulant state with dampened fibrinolysis at HA.^13^, ^14^, ^15^ Chronic hypoxia, by way of endothelial activation and inflammatory response, may also predispose to VTE at HA.^16^, ^17^, ^18^, ^19^ We have reported earlier that thrombosis at HA is regulated by a complex network of coagulatory and inflammatory processes, linked through Hypoxia-inducible factor-1α (HIF1α).^18^^,^^20^

The numbers of lowlanders visiting HA for work and leisure activities are ever-increasing, driving the imperative to elucidate the role of HA as a risk factor for thrombosis. Few people stay at extreme high altitudes (EHA>18,000ft/5,500m) for long; mountaineers usually not spending more than 7-11 days at these altitudes. Amongst sojourners, only soldiers in the Himalayas have uninterrupted stay of 3-4 months at altitudes >15,000ft (4,572m).^21^ Large-scale prospective studies of healthy lowlanders developing thrombosis at HA are conspicuous by their absence; the logistical burden of conducting such studies being extremely challenging.

This prospective study was conducted to evaluate the epidemiology and pathophysiology of thrombotic disorders in healthy soldiers during an uninterrupted stay of 3-4 months at altitudes higher than 15,000ft (4,572m).

## Methods

A cohort of healthy male soldiers who ascended, in the course of routine duty, from June 2012 to June 2014, to a HA area in the Western Himalayas was prospectively observed from sea level through an uninterrupted sojourn of 3-4 months at >15,000ft (4,572m). The study was registered with the Indian Armed Forces Medical Research Committee (Project No 4143/2011) and conducted in accordance with the ethical standards of the Indian Council of Medical Research. Informed consent was obtained as per the Declaration of Helsinki.

### Definitions

In this report, altitudes of 12-15,000ft (3,658-4,572m) are called HA1 and altitudes >15,000ft (4,572m), HA2. Index cases (ICs) are subjects diagnosed with thrombotic events, arterial or venous, at HA. The term ‘screening’ implies detailed clinical examination and collection of blood samples for on-site tests and storage.

### Screening schedule and diagnostic criteria

Subjects were screened thrice, the first screening being at near-sea-level (1,072ft;327m), the second between 6-12 weeks stay at HA1 and the third subsequent to descent from HA2 after a 3-4 months’ uninterrupted sojourn there. The third screening was done at 12,000ft (3,658m), within 24 hours of descent, in all healthy subjects. The ICs were air-evacuated, from HA2 locations, directly to tertiary care centres at near-sea-level, within twenty-four hours of occurrence of the event. All cases where a thrombotic episode was clinically suspected were confirmed by imaging modalities including colour doppler, MRI and CT scan. In ICs, the third screening and detailed evaluation was conducted at these centres along with management of the thrombotic ailment.

### Sampling and inclusion/exclusion criteria

No previous study could be found, which provided estimates of the incidence of thrombosis at various body sites in temporary residents at HA. Hence, a convenience sample of all members of an operational military unit ascending to HA in the course of routine duties were included as subjects in this study. Nine hundred and sixty subjects were included and screened at sea level. As per extant organizational policies those with systemic hypertension, metabolic syndrome, bronchial allergy, musculoskeletal disorders, pulmonary artery hypertension, varicose veins, previous VTE/ varicocele/ portal vein thrombosis, any history of high-altitude illnesses (except acute mountain sickness) or any systemic ailment/chronic therapy were considered unfit for prolonged stay at HA and excluded from the study. Subjects who developed any acute/chronic HA illness (other than acute mountain sickness) or sustained systemic hypertension with stay at HA were excluded from the study and referred for management to tertiary care centres in the plains.

### Selection of comparison group (CG)

Since the ICs were at different locations at HA2, three study subjects per IC, who had ascended to and were staying at the same HA2 location formed the CG (Control). At locations with more than four subjects including the IC, three CG subjects were chosen by random sampling using random number tables. At two locations where only three individuals were present (including the ICs) only two CG subjects per IC were available. Thus, 43 study subjects formed the nested CG.

### Subjects, ascent and sojourn profile

**Figure 1:**
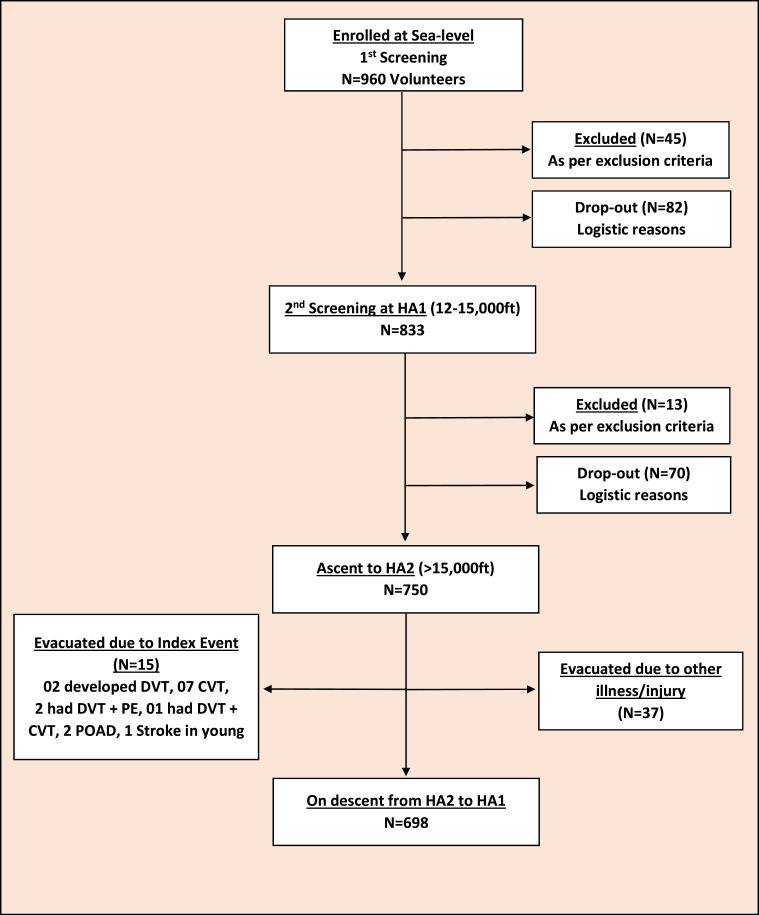
Consort diagram: Enrollment and populations for analysis. HA1 – High Altitude 1 (12,000-15,000ft), HA2 – High Altitude 2 (>15,000ft).

In the plains, 960 subjects were screened of which 833 subjects ascended to HA1 (Figure 1), where they acclimatized and trained as per existing organizational protocols (Supplement 1). Of these, 750 ascended to HA2, including 651 to extreme high altitude (EHA>18,000ft/5,500m) (Video 1). As this longitudinal study was conducted on soldiers in the course of routine duties at high altitudes, there were limitations in capturing all subjects at various ‘Screening’ points due to inescapable logistic, administrative, operational, and technical (equipment malfunction on-site) reasons. While at HA2, 52 subjects with various ailments were air-evacuated to tertiary care hospitals in the plains for detailed evaluation and management. Those found to have suffered vascular thrombosis, the ICs (n-15), were treated as per institutional policies with anticoagulation and supportive care. They were not allowed to return to HA. Healthy subjects remaining at HA2 (n-698) underwent the third screening on descent to HA1.

### Methodology

Health care providers from six hospitals (three at sea level and three at HA) were involved in screening, sample collection, and treatment. The subjects were screened by a team of trained doctors and paramedics at all altitudes.

Blood samples were tested on-site for complete blood counts and coagulation-screen parameters (Prothrombin Time, Thrombin Time, activated Partial Thromboplastin Time and Fibrinogen). Additionally, samples for the entire cohort were stored as whole blood, platelet-poor plasma, and serum at -80˚C. Molecular markers of coagulation, fibrinolysis, platelet activation, endothelial activation, and inflammation were tested in a referral laboratory at near-sea-level (Supplement 2). Genotyping was done for 17 single nucleotide polymorphisms (SNPs) associated with thrombotic disorders. All ICs were screened for thrombophilia (Protein-S activity, levels of Anti-thrombin, and Activated Protein C Resistance) after cessation of anticoagulant therapy, 6-9 months post-event.

### Statistical analysis

JMP 15.0 was used for statistical analysis. The incidence of thrombotic events was calculated per hundred thousand person-years. Since the analysis involved estimation of rates of venous and arterial thrombosis during defined period of observation where each person contributed differently and was followed for different lengths of time, it is measured in years of observation time per person. Thus, person-time is an estimate of the actual time-at-risk in years that all participants contributed to the study. Continuous data variables were assessed for normal distribution. Parametric tests were applied for normally distributed data and non-parametric tests for data not normally distributed. A p-value of less than 0.05 was considered statistically significant. Data are presented as mean plus/minus standard deviation (median; range). Violin and scatter plots have been used to depict the distribution of molecular markers over the different phases of the study. Fisher's exact test was used to seek the significance of the difference in frequency of distribution of SNPs between groups, and the Odds Ratio was calculated for an association of SNPs with manifestation. The incidence of thrombosis was calculated in the entire cohort, whereas the molecular studies were limited to the ICs and the CG.

### Role of funding source

The funders had no role in the design, data collection, analysis, and interpretation, or preparation of this manuscript.

## Results

Fifteen subjects developed thrombotic events at HA2, twelve developed venous and three, arterial thrombosis, respectively. In this study, the incidence of venous thrombosis was 5,926/10^5^ person-years; 3,951/10^5^ person-years for cerebral venous thrombosis (CVT), and 2,469/10^5^ person-years for deep vein thrombosis with or without pulmonary thromboembolism (DVT±PTE). The incidence of arterial thrombosis was 1,482/10^5^ person-years; 494/10^5^ person-years for stroke, and 988/10^5^ person-years for peripheral arterial occlusive disease (PAOD) (Supplement 3). Vascular thrombotic episodes occurred only in the period of 3-4 months of uninterrupted stay above 15,000ft, none occurring during the remaining 20-21 months at HA.

The ICs were older than the CG (32±5.9y vs 27.4±5.7y; p-0.011). They also had higher body-mass index (23.8 Vs. 22.3kg/m^2^, p-0.018) and waist circumference (88.3 Vs. 81.4cm, p-0.01) at baseline. The groups were matched for altitude of stay; mean altitude in ICs and CG being 19,262±1,591ft (5,871±485m) and 18,902±1,573ft (5,761±480m) (p-0.32), respectively. The duration of stay at HA2 was significantly shorter in ICs (Median/Interquartile range: 80.5/70-108 vs 109/97.5-112.75) since they were evacuated to near-sea-level on occurrence of ailment. The prevalence of alcohol consumption and smoking was comparable in both groups on univariate analysis. On multivariate analysis, body-mass index, waist circumference, and age were not significantly different between the two groups.

The clinical details of ICs are tabulated in Table 1. Cerebral venous thrombosis was noted in seven subjects at HA2 occurring between 40-109 days of stay. Arterial thrombosis resulted in right middle cerebral artery (MCA) stroke in one case and peripheral arterial occlusive disease (PAOD) in two cases. One subject with PAOD had concurrent superior mesenteric artery thrombosis. He succumbed to sepsis following above-knee amputation of the right leg and small gut resection for gangrene. Three of eight cases (37.5%) of CVT and three of five (60%) of VTE had thrombophilia (Table 1). Eight of the ICs (53.3%) had elevated Factor VIII, and four (26.7%) elevated von Willebrand factor levels; ten (66.7%) had hyperhomocysteinaemia (serum homocysteine > 20 µmol/L; mean: 33.48±17.89), and four (26.7%) had low serum iron, ferritin, and high iron-binding capacity in the absence of anaemia. Of those with activated protein-C resistance (APCR), one was heterozygous for Factor V Leiden (FVL).

**Table 1:** Clinical details and risk factors for thrombosis in index cases.

Case Number	Age (Yrs)	Altitude (ft)	Day at HA2 symptom onset	Symptoms at onset	Radiological findings at presentation	Clinical/ imaging status 6-18 months after the event	Risk Factors*
1	36	16,645	101	Sudden onset focal, sensorimotor deficit of the left upper limb.	Thrombosis of the Superior Sagittal Sinus, with right frontal & left frontoparietal cortical bleed. Atretic left transverse sinus on CT scan & digital subtraction angiography.	Headache, disturbed sleep & asthenia. Evidence of recanalized Superior Sagittal Sinus. Atretic left transverse sinus. No parenchymal abnormality on MRI brain & MRV.	Obese, ↓ Functional Protein S, ↓Ferritin, HHcy, ↑vWF
2	21	20,410	79	Occasional headache from 10^th^ days at altitude. Sudden onset, severe global headache, giddiness & vomiting on 79^th^ day.	Thrombosis of the right & left transverse & sigmoid sinuses and torcula extending into the right Internal Jugular vein on MRI brain & MRV.	No symptoms. Sinuses recanalized on MRI brain & MRV.	↓Ferritin, HHcy, ↑ F VIII
3	31	17,800	109	Right hemi-cranial headache of increasing severity, giddiness & recurrent vomiting for 03 days.	Right transverse & sigmoid sinus thrombosis with hemorrhage & infarcts in the right temporoparietal cortex & sub-cortical white matter on MRI brain & MRV.	No symptoms. Chronic thrombus of right transverse & sigmoid sinuses. Gliosis & encephalomalacia right middle & inferior temporal gyri with dilation of the temporal horn of lateral ventricle on MRI brain & MRV.	Overweight, ↑ F VIII
4	28	19,720	61	Headache, giddiness, and malaise for 03 days, followed by one episode of Generalized Tonic-Clonic Seizure on day 64.	Thrombosis of the Superior Sagittal Sinus on MRI brain & MRV.	Occasional headache. Sinus recanalized on MRI brain & MRV	Overweight, HHcy
5	41	19,600	107#	Intermittent headache for 01 day and one episode of vomiting after 4 hours of downhill walking.	Thrombosis of the Superior Sagittal, right Transverse & Sigmoid cerebral sinuses on MRI brain & MRV.	No symptoms. Recanalized appearance of Superior Sagittal Sinus. Chronic thrombosis of the right Transverse & Sigmoid Sinuses on MRI brain & MRV.	Smoking, HHcy, ↑ F VIII & vWF
6	37	20,410	67	3 episodes of slurred speech & focal sensorimotor deficit involving the right arm and face through one night.	Thrombosis of the Superior Sagittal Sinus with bilateral infarction within the superior & inferior frontal gyri. Hypoplastic right transverse sinus on MRI brain & MRV.	Lost to follow-up	
7	33	20,525	40#	Headache every 3-4 days starting approx. on the 40th day, at altitude, continuing through another 72 days at HA2 and on the descent to 12,000 feet.	Thrombosis of the left Transverse & Sigmoid sinuses, extending into the left Internal Jugular Vein on CECT head & CT Venography	Occasional headache. Chronic thrombosis of the left Transverse & Sigmoid sinuses on MRI brain & MRV.	Overweight, APCR, HHcy
8	21	21,000	82	Sudden onset pain with mild swelling & tenderness right calf for 03 days	No evidence of DVT on CDFI	6 months later, CDFI showed recanalized thrombus of the right Popliteal & Post Tibial veins. MRI brain &MRV showed – Thrombosis of left Transverse & Sigmoid sinuses extending to the Jugular bulb. 12 months later, the subject was asymptomatic. CDFI showed recanalized leg veins. MRV showed attenuated proximal left Transverse sinus with collateral vessels forming distal Transverse and Sigmoid sinuses.	APCR (FVL Heterozygous), ↓ Ferritin, HHcy, ↑ F VIII
9	29	15,632	61	Discomfort right groin for 07days followed by pain & swelling of the right calf.	Thrombosis of the right Superficial Femoral & Popliteal veins on CDFI.	Clinical and doppler features of chronic DVT, right leg on CDFI.	APCR, HHcy, ↑ F VIII
10	37	18,700	19	Progressive pain, swelling & tenderness of left calf & thigh for 03 days.	Thrombosis of the left distal Superficial Femoral, Popliteal, Anterior Tibial & Posterior Tibial Veins on CDFI. Thrombus involving the descending branch of the left Pulmonary artery on HRCT chest.	Clinical features of chronic DVT. Chronic thrombus of the left Popliteal & Post Tibial veins with recanalization on CDFI. Parenchymal nodule in the superior segment of the lower lobe of left lung on HRCT chest.	Overweight, APCR, HHcy, ↑ F VIII & vWF
11	38	19,600	58	Fever for 01 day, followed by dry cough and progressive DOE for 10 days; NYHA Cl IV on day of reporting.	Partial thrombus of right Popliteal vein on CDFI. PTE of right main & upper Pulmonary arteries on HRCT Chest.	Dyspnea on exertion, NYHA Cl I. Segmental perfusion defect in the apical & anterior segments of right upper lung lobe seen on lung perfusion scan.	Smoking, overweight, ↓Ferritin, HHcy, ↑ F VIII & vWF
12	31	21,000	73	Asthenia & backache for 03 days, followed by swelling & pain of the left thigh & calf for 02 days	Thrombosis of the left Common & External Iliac, Common Femoral & Great Saphenous veins on CDFI.	Clinical Features of Chronic DVT left lower limb. Chronic thrombosis of the left External Iliac, Common Femoral, and left Great Saphenous veins on CDFI.	
13	34	18,685	22#	Chronic headache with 04-05 episodes of nausea, vomiting & dysarthria lasting a few minutes over next 76 days of sojourn at HA2	Ill-defined focal lesion (MCA territory) on MRI Brain	Heaviness of head. Foci of gliosis in the deep & peri-trigonal white matter, right cerebral hemisphere on MRI brain.	Overweight, Family H/o CAD, HHcy
14	28	20,405	64	Acute incapacitating cramps both lower limbs with associated numbness. Discoloration & pain of the left forefoot.	Angiography showed Echogenic content & no flow in the left Superficial Femoral Artery and Bilateral Popliteal, Ant & Post Tibial arteries suggestive of PAOD, on angiography	Progressively decreasing claudication distance. Wall thickening & stenosis of left Common & External Iliac, Common Femoral & Superficial Femoral arteries. Echogenic content in distal Superficial Femoral. Reduced flow in the Popliteal artery. No flow in anterior & posterior Tibial arteries. Stenosis proximal to right anterior & posterior Tibial arteries on CDFI.	Smoking, overweight, ↑ F VIII
15	35	18,685	74	Acute abdominal pain	Superior mesenteric artery thrombosis with PAOD right lower limb on angiography	Died due to complications of small gut resection (septicemia).	Smoking, overweight

⁎ Yrs – years; Ft – feet; HA2- High altitude phase 2, EHA – Extreme High Altitude; Overweight – BMI 23-24.9 Kg/m2; Obese – BMI≥ 25 Kg/m2; CT -Computed tomography; MRV – Magnetic resonance venography; MRI -Magnetic resonance imaging; ↓ - decrease; ↑ - increase; HHcy – Hyperhomocysteinemia; vWF- Von Willebrand Factor; FVIII – Factor Eight; APCR- Activated Protein C Resistance; DVT- Deep Vein Thrombosis; CDFI – Color doppler flow imaging; HRCT – High resolution computed tomography; PTE – Pulmonary thromboembolism; NYHA – New York Heart Association; MCA -Middle Cerebral Artery; CAD – Coronary Artery Disease; PAOD – Peripheral Arterial Occlusive disease

The haematological and coagulation profile of the ICs and CG over the duration of the study is presented in Table 2. The increase in haemoglobin (Hb) with gain of altitude was significantly lesser in ICs than in CG. ICs had a rise in total leucocyte count (p = 0.0013) and granulocytes (p<0.001) but had lower lymphocyte counts (p<0.001) with increasing altitude. The monocyte counts were comparable at the second screening (at HA1), but the ICs had significantly lower monocyte counts (p<0.0001) post-event. Platelet counts were not significantly different as a function of group or altitude. Prothrombin time (PT) and activated partial thromboplastin time (aPTT) shortened significantly in ICs and CG on ascent to HA1 (p = 0.017), only to return to baseline values on descent from HA2 (p = 0.022) (Supplement 4).

**Table 2:** Hematological and coagulation profile of the cases and comparison group in the table across different Screening points in the study.

Parameter	Screening	N		Mean		Median		Std Dev		Min		Max		p value
		Controls	Cases	Controls	Cases	Controls	Cases	Controls	Cases	Controls	Cases	Controls	Cases	
**Hemogram**														
Hb	Screening 1*	22	8	12.82	13.01	12.25	13.10	2.90	3.64	7.30	7.50	19.20	19.80	0.8967
	Screening 2*	22	7	18.20	16.23	18.29	16.40	1.31	0.73	14.90	15.20	20.41	17.00	**<0.0001**
	Screening 3*	22	9	18.35	15.61	18.65	15.80	1.77	0.83	13.80	14.20	21.40	16.40	**<0.0001**
Hct	Screening 1*	22	8	38.29	39.73	35.60	42.30	7.96	10.86	23.80	22.60	56.80	57.50	0.739
	Screening 2*	22	7	39.23	36.39	39.75	34.60	6.77	9.10	29.75	27.10	58.60	55.10	0.4673
	Screening 3*	22	0	56.29	.	57.90	.	5.78	.	41.40	.	63.60	.	
WBC	Screening 1*	22	8	6.97	6.17	6.70	6.48	2.29	2.11	3.58	2.99	10.41	8.98	0.3887
	Screening 2*	22	7	7.52	9.11	7.33	8.99	1.78	1.42	4.59	7.24	10.80	11.34	**0.0309**
	Screening 3*	22	9	6.89	9.94	6.35	9.10	2.18	1.92	4.10	7.20	13.40	13.00	**0.0013**
Poly	Screening 1*	22	8	57.45	59.16	57.45	59.70	7.97	5.16	44.60	50.60	72.00	67.20	0.499
	Screening 2*	22	7	64.43	69.57	63.35	71.40	5.48	6.41	58.00	61.80	75.80	77.10	0.0885
	Screening 3	22	9	49.67	72.78	47.45	73.00	8.49	8.17	37.20	62.00	66.90	82.00	**≤0.0001**
Lympho	Screening 1*	22	8	37.54	35.46	35.90	33.65	7.76	4.78	24.20	29.80	51.40	44.60	0.3896
	Screening 2*	22	7	32.96	27.7	32.80	26.60	5.15	5.38	22.20	21.10	39.60	33.50	**0.0467**
	Screening 3*	22	9	38.40	32.76	40.55	25.00	7.70	7.57	23.40	17.00	50.10	35.00	**0.0007**
Mono	Screening 1	22	8	5.01	5.38	4.65	4.60	1.32	2.39	3.30	3.00	9.00	10.70	0.9438
	Screening 2	22	7	2.61	2.73	2.10	2.00	1.09	1.31	1.60	1.80	4.90	4.70	0.7546
	Screening 3	22	9	11.15	1.67	11.25	1.00	4.56	0.87	1.50	1.00	21.00	3.00	**≤0.0001**
THR	Screening 1	22	8	314.27	348.50	276.50	359.50	180.58	148.55	91.00	124.00	955.00	525.00	0.4118
	Screening 2*	22	7	293.41	258.29	291.50	244.00	95.31	100.27	94.00	160.00	463.00	466.00	0.4336
	Screening 3*	9	0	274.11	.	291.00	.	42.94	.	218.00	.	326.00	.	
**Coagulogram**														
PT	Screening 1	35	8	13.85	14.51	13.60	13.60	1.83	2.54	11.20	11.90	18.20	18.60	0.6734
	Screening 2*	35	8	12.73	12.93	12.70	12.45	0.84	1.21	11.20	11.90	15.00	15.10	0.6695
	Screening 3	35	5	13.95	15.97	13.10	16.25	2.84	1.76	10.80	13.00	27.10	17.40	0.0231
APTT	Screening 1	35	8	33.65	35.25	34.40	35.15	6.65	6.04	4.80	25.80	51.30	46.50	0.5222
	Screening 2*	35	8	27.56	26.43	27.00	25.05	3.16	3.03	20.50	23.00	35.20	31.10	0.3627
	Screening 3	35	0	32.26	.	31.20	.	5.49	.	24.10	.	53.50	.	

Acronyms used: (N – number in each group; Std Dev – Standard Deviation; Min – Minimum, Max – Maximum; Hb – Hemoglobin (g/dl); Hct – Hematocrit (%); WBC – white blood cell count (x10^9^/µL); Poly – Polymorphs (%); Lympho – Lymphocytes (%); Mono- Monocytes (%); THR – platelet count (x10^9^/µL); PT – prothrombin time (seconds); APTT – activated partial thromboplastin time (seconds).

Legend: Asterisk (*) on phases indicate normal distribution of the parameter, lack of asterisk indicates not normal distribution; Bold in ‘p’ values indicate statistically significant; Underlined ‘p’ values indicate analysis by Wilcoxon test, those which are not underlined were analyzed by student's t test)

**Figure 2:**
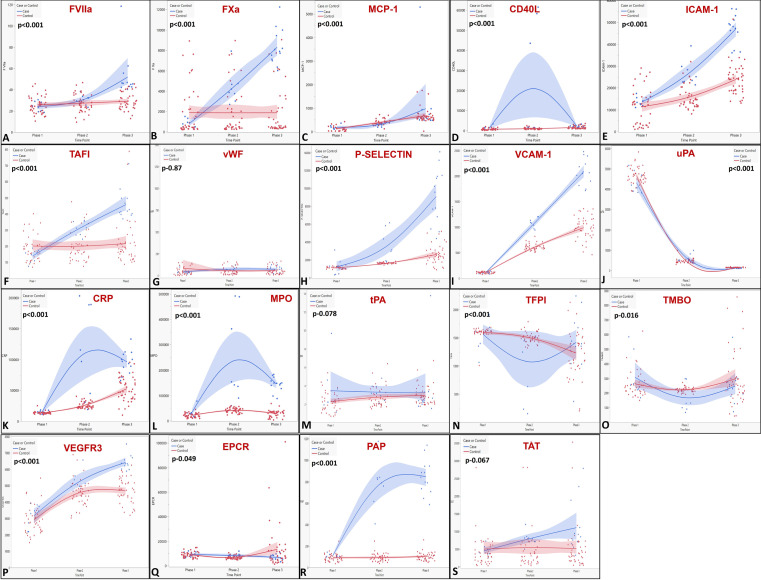
Scatter plot showing the trend of the molecular markers in cases and controls across the three Screenings in the study for (A) Factor VII activated (FVIIa; mU/ml) (B) Factor X activated (FXa; mU/ml) (C) Monocyte chemoattractant protein-1 (MCP-1; pg/ml) (D) CD_40_ ligand (CD40L; pg/ml) (E) Intercellular Adhesion Molecule 1 (ICAM-1; pg/ml) (F) Thrombin activated fibrinolysis inhibitor (TAFI; pg/ml) (G) Von Willebrand factor (vWF; mU/ml) (H) P-Selectin (pg/ml) (I) Vascular cell adhesion molecule type 1 (VCAM-1; pg/ml) (J) Urokinase type plasminogen activator (uPA; pg/ml) (K) C reactive protein (CRP; pg/ml) (L) MPO pg/ml (M) tissue plasminogen activator (tPA; pg/ml) (N) Tissue factor pathway inhibitor (TFPI; pg/ml) (O) thrombomodulin (TMBO; pg/ml) (P) Vascular endothelial growth factor receptor 3 (VEGFR3; pg/ml) (Q) Endothelial protein C receptor (EPCR; pg/ml) (R) Plasmin antiplasmin complexes (PAP; pg/ml) (S) Thrombin antithrombin complexes (TAT; pg/ml) (The X-axis represents the three Screenings in the study (Screening1  (Phase 1) – sea level; Screening 2  (Phase 2) – High altitude 1; Screening 3 (Phase 3) – at High Altitude 1 after descent form High Altitude 2. The values of the various markers for each participant are represented on the Y-Axis). The curves represent the relation between the different Screenings for the cases and comparison group (controls) separately, with the shaded area representing the confidence of fit. The p values represent the ANOVA).

**Figure 3:**
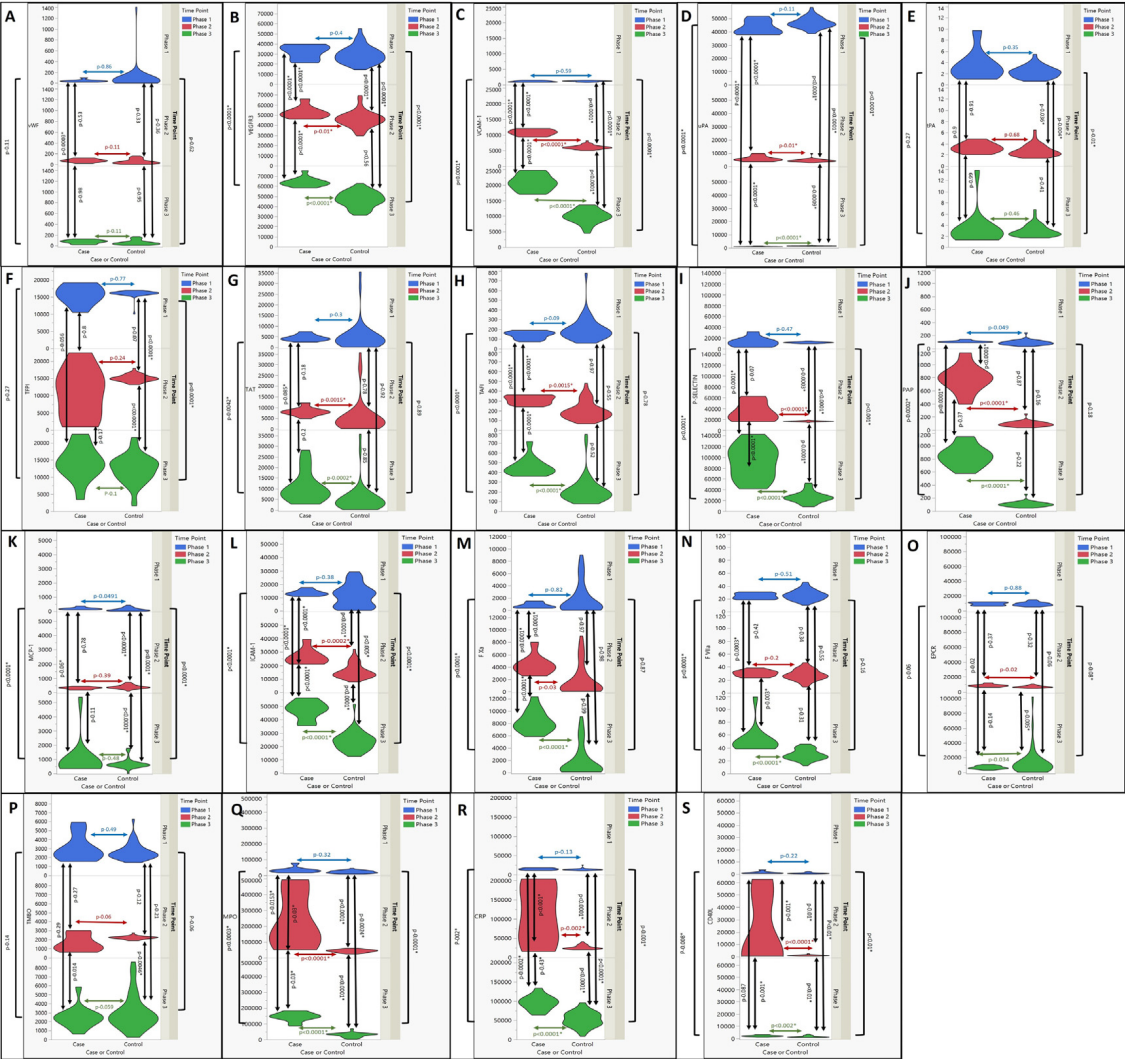
Violin plots showing the molecular markers in cases and controls across the three Screenings in the study for (A) Von Willebrand factor (vWF; mU/ml) (B) Vascular endothelial growth factor receptor 3 (VEGFR3; pg/ml) (C) Vascular cell adhesion molecule type 1 (VCAM-1; pg/ml) (D) Urokinase type plasminogen activator (uPA; pg/ml) (E) tissue plasminogen activator (tPA; pg/ml) (F) Tissue factor pathway inhibitor (TFPI; pg/ml) (G) Thrombin antithrombin complexes (TAT; pg/ml) (H) Thrombin activated fibrinolysis inhibitor (TAFI; pg/ml) (I) P-Selectin (pg/ml) (J) Plasmin antiplasmin complexes (PAP; pg/ml) (K) Monocyte chemoattractant protein-1 (MCP-1; pg/ml) (L) Intercellular Adhesion Molecule 1 (ICAM-1; pg/ml) (M) Factor X activated (FXa; mU/ml) (N) Factor VII activated (FVIIa; mU/ml) (O) Endothelial protein C receptor (EPCR; pg/ml) (P) thrombomodulin (TMBO; pg/ml) (Q) Myeloperoxidase (MPO; pg/ml) (R) C reactive protein (CRP; pg/ml) (S) CD_40_ ligand (CD40L; pg/ml) (Index Cases and controls are separated on the X-Axis. On Y-axis on the left, the values of the various markers for each individual are marked and on the right the Y-Axis represents the three Screenings in the study (Screening1 – sea level; Screening 2 – High altitude 1; Screening 3 – after descent from High altitude 2 at High altitude1). The violin plots (scaled for equal width) represent the distribution of the molecular markers in each group (cases and controls) at different Screenings separately. The p values indicating statistical difference across groups have been calculated using Fit Y by X analysis (Student's t-test for variables with normal distribution and Wilcoxon/ Kruskal Wallis test for variables without normal distribution).

The landscape of molecular markers in subjects through the study is depicted in Figures 2 and 3. Procoagulant factors (VIIa, Xa, and thrombin activatable fibrinolysis inhibitor) significantly increased in ICs at HA. The naturally occurring anticoagulants (thrombomodulin and tissue factor pathway inhibitor) had a decremental response in both groups which, however, was not statistically significant. Of the markers for fibrinolytic activity, urokinase-type plasminogen activator (uPA) had a decremental response whereas, tissue plasminogen activator (tPA) remained unchanged. (Figures 2, 3) (*Supplement 4*).

Markers of inflammation, platelet activation and endothelial activation (CD40L, C-reactive protein [CRP], myeloperoxidase [MPO], vascular endothelial growth factor receptor 3 [VEGFR-3], intercellular adhesion molecule-1 [ICAM-1], vascular cell adhesion molecule-1 [VCAM-1], and P-Selectin) increased significantly and steadily with altitude (p<0.001) (Figures 2, 3) This change was noted in both groups, being significantly higher in ICs (p<0.0001). Interestingly, MPO, monocyte chemoattractant protein-1 (MCP-1) and plasmin-α2-antiplasmin complex (PAP) levels were significantly higher at sea-level in IC's compared to the CG, in addition to that in HA (Supplement 4).

The analysis of genes for prothrombin, tissue factor pathway inhibitor (TFPI), endothelial protein C receptor (EPCR), and angiotensin-converting enzyme (ACE) were found monomorphic in both ICs and CG. Contingency tests for thrombosis-associated SNPs were significantly higher in ICs for PAI1-6754G/5G and MTHFR677C/T (p<0.0001) (Supplement 4). The odds ratio for MTHFR677C/T mutation in ICs was significant (χ2; p<0.001).

## Discussion

Exposure of lowland humans to HA is progressively increasing, given international geopolitical concerns, sporting activities, tourism, and occupational requirements. HA poses a peculiar challenge to humans, wherein an overdrive of physiological compensation may result in pathological consequences, such as thrombotic events, often disastrous, in healthy individuals.

Sojourners at HA experience chronic hypoxia and those with sleep disordered breathing might well have superimposed chronic intermittent hypoxia. Chronic hypoxia and inflammation are linked at the molecular level, in diseases such as obesity, cancers, acute lung injury and infections.^16^ Our earlier work has also demonstrated the link from inflammation to thrombosis via the NLR family pyrin domain containing 3 (NLRP3) inflammasome.^18^

Thrombosis at HA accounts for significant morbidity and mortality even in healthy individuals^4^^,^^22^ but a search of literature revealed only short-exposure, small sample size studies, and retrospective articles/case reports on this subject.^23^, ^24^, ^25^ A study was, therefore, warranted to describe the epidemiology of vascular thrombosis at extremes of altitude, to address this unmet need. This comprehensive study in healthy subjects focused on thrombotic events through 24 months of stay at HA, including an uninterrupted stay of three to four months at HA2.

The incidence of thrombotic events at HA2 found in this study is several orders of magnitude greater than reported in the general population at sea level.^2^^,^^26^ Besides, the incidence reported in the general population includes both healthy and sick groups, whereas in the present study, it refers only to a healthy population. The incidence of VTE of 2,469/10^5^ person-years is two orders of magnitude greater than the estimate of 0.7-2.69 per thousand population at near sea level. The incidence of CVT of 3,951/10^5^ person-years (08/750=1.07%, i.e., 1.07 × 10^4^/million) is several orders of magnitude greater than the estimate of 3-4 per million reported in the general population. Similarly, the incidence of Stroke and PAOD is a couple of orders more than the reported incidence of 8.07/10^5^ and 14/10^5^ person-years, respectively, at near sea level.^27^^,^^28^

Various authors have reported alterations in Hb/haematocrit, platelets, and white cells, in transient acclimatized low landers at HA, some reporting a rise and others a decline. However, the study designs, time points, and altitudes of sample collection in these studies were not comparable.^29^^,^^30^ In our study, ICs showed lower haemoglobin and haematocrit than the CG. It has been postulated that anaemia results in poor deformability of the RBCs, increasing blood viscosity, thereby predisposing to thrombosis.^31^^,^^32^

Neutrophils and monocytes, have been reported to play a pivotal role in thrombus causation.^33^^,^^34^ Interestingly, we noted that the neutrophil counts were significantly higher in ICs at HA, being more marked after the index event.^33^ Lower platelet numbers noted in ICs could be attributed to ongoing thrombosis, while raised levels of P-Selectin and CD40 ligand suggest functional platelet activation.^15^

Markers of endothelial dysfunction (ICAM1, VCAM, VEGFR3, thrombomodulin) and inflammatory response (CRP, MPO) were also noted to progressively increase with rising altitude in both groups, more prominently so in ICs and has been reported earlier by us.^20^ Interestingly MPO, MCP-1 and PAP were also significantly higher at sea-level in the IC's in addition to in HA (Supplement 4). This raises the possibility for considering these biomarkers as predictors of thrombosis. However, this would need to be established in a prospective, adequately powered study. CD40L promotes expression of adhesion molecules such as P-selectin by activated platelets, resulting in enhanced platelet-endothelial interaction during thrombogenesis.^20^^,^^35^ Of note, it has been reported that raised levels of markers of inflammation and endothelial dysfunction in cases of VTE, may remain elevated for periods up to five years.^36^

We had previously reported that hypoxia-inducible factor 1α HIF (HIF-1α) and inflammatory response mediated by NLRP3 inflammasome complex, are crucial determinants of thrombotic events in hypoxic conditions.^18^ These pathways could trigger a procoagulant state, suggested by the increase in molecular markers of coagulation, inflammation and endothelial and platelet activation, in the present study. Reactive oxygen species formed secondary to hypobaric hypoxia are believed to be responsible for initiating the inflammatory cascade and vascular endothelial dysfunction.^37^

In the present study, the picture overall, is of a procoagulant state, with increased coagulation (Factors VIIa and Xa) and decreased naturally occurring anticoagulant activity (thrombomodulin and TFPI) at HA2, which was more pronounced in ICs. In contrast, the CG displayed an increase in fibrinolytic activity, which is antithrombotic and protective. Exercise is known to induce a pro-coagulant laboratory picture. However, both the IC and CG being co-located performed the same tasks and similar levels of activity. Hence, it is unlikely that exercise confounded the comparison between groups in this study. On the other hand, the local topography and extreme altitudes at which the soldiers were located, enforced restricted activity on the entire cohort under study.

The role of MTHFR677C/T SNP association with thrombosis is not yet established.^38^ PAI-1-6754G/5G and MTHFR677C/T were over-represented in ICs compared to CG, which is concordant with our group's earlier report of this association with VTE among Indian subpopulations.^39^^,^^40^ However, in the absence of other thrombophilic defects, cardiovascular and metabolic disorders, the PAI-1-6754G/5G variant has a poor association with thrombosis in the healthy population.^41^ Genes for Factor V Leiden, prothrombin, TFPI, EPCR, and ACE were found monomorphic in both CG and ICs suggestive of partial and restrained penetrance of these genetic mutation for thrombotic manifestation in Indian subpopulations.

All thrombotic events, in this study, occurred at altitudes >15,000ft (4,572m) among healthy subjects. Interestingly, arterial events (n=03) occurred only at altitudes >18,000ft (5,500m). It would, thus, appear that accentuated chronic hypoxia at >15,000ft (4,572m) could be an independent risk factor for thrombosis, triggering inflammation with endothelial dysfunction resulting in enhanced coagulation. Combined with dampened fibrinolysis, this could cause thrombotic events, with significant attendant morbidity and mortality.

Occurrence of VTE in healthy soldiers at HA, as evidenced by our study, highlights the need for stringent screening to rule out any underlying medical disorders prior to ascent to extreme altitudes. This might be even more relevant for the general population.

The study's major strength was the successful monitoring for ailments at HA in healthy subjects over prolonged periods of stay at >15,000ft (4,572m). Health care providers from three hospitals at sea-level and three at HA, were involved in the study.

Conducting studies at these altitudes is costly, labour-intensive, logistically challenging, and compounded by inclement weather conditions. The terrain remains equally challenging for researchers and one of our team members suffered High Altitude Cerebral Edema (HACE) at an altitude of 13,500ft (4,115m). Others had episodes of acute mountain sickness. Fortunately, all recovered completely with therapy and descent to lower altitude.

The major limitations of this study were the absence of female subjects, limited age group, absence of family screening to establish inherited thrombophilia and inclusion of only healthy subjects as the study group. Also, since this longitudinal study was conducted on soldiers in the course of duties at high altitudes, there were limitations to capturing all subjects/data at various ‘Screening’ points due to inescapable logistic, administrative, and technical (equipment malfunction on-site) reasons. Hence, despite our best efforts, the numbers of subjects in whom data for various parameters could be captured, vary at different screening points.

## Conclusion

The ICs were noted to have a prothrombotic state with suppressed naturally occurring anticoagulants, dampened fibrinolysis, endothelial activation, platelet activation and raised proinflammatory markers. The incidence of clinically manifest thrombotic events, venous more than arterial, at HA2 (altitudes >15,000ft/4,572m) among the healthy subjects of this study was markedly higher than that reported at near sea level. Altitude >15,000ft (4,572m) may be an independent risk factor for thrombosis, even in healthy subjects.

## Contributors

VN conceptualized and designed the study. VN, SPS, MZA, UY, VS, AP, TC, VB, VG, SP, SG, MGV, RV, KK, RA, PS, NB, TDK, WTW, SAB, RD, PG were involved with patient management, sample collection, data collection and data entry.

VN, SPS, UY, MZA, SP were responsible for digitalization of records, statistical analysis, and manuscript preparation. All authors have read and agreed to the published version of the manuscript.

## Data sharing statement

A non-identifiable copy of the dataset will be loaded onto https://www.researchgate.net/publication/360453713_Repository_HA-VTE_study.

## Dedication

This original work is dedicated to Late Col (Dr.) Prosenjit Ganguli who contributed significantly to this work prior to his untimely demise on 07th Nov 2020.

## Declaration of interests

The authors have no relevant conflicts of interest to declare.

## Appendix Supplementary materials

Image, application 1
