# Supplementary material for: Epidemiology and pathophysiology of vascular thrombosis in acclimatized lowlanders at high altitude: A prospective longitudinal study
Source: Lancet Reg Health Southeast Asia. 2022 Jun 9;3:100016. doi: 10.1016/j.lansea.2022.05.005 (PMC10306047; doi:10.1016/j.lansea.2022.05.005)
Supplement: Supplementary file 1 [file mmc1.docx]

| **S No** | **SUPPLEMENT** | **TITLE** | **PAGE NO** |
| --- | --- | --- | --- |
| **1** | **SUPPLEMENT 1** | **Ascent and sojourn profile of study subjects at HA/EHA** | **2** |
| **2** | **SUPPLEMENT 2** | **Methodology** | **3** |
| **3** | **SUPPLEMENT 3** | **Details of the thrombotic events in the study cohort** | **7** |
| **4** | **SUPPLEMENT 4** | **The details of the (A) Coagulogram (B) Hemogram and (C) Molecular markers** | **8** |
| **5** | **SUPPLEMENT 5** | **Contingency test for thrombosis associated SNPs between patients and study control subjects** | **13** |

**SUPPLEMENT 1. ASCENT, SOJOURN AND SCREENING SCHEDULE OF THE COHORT DURING THE PERIOD OF STUDY.**

3658m

3658-4572m

Study cohort at HA1

2700-3658m

3^rd^ Screening

screening

1^st^ Screening

Ascent & Acclimatization

Half the study cohort at HA2

Balance of study cohort at HA2

3-4 months

3-4 months

4 months

2 Wks

4572-6700m

NSL

2^nd^ Screening

Screening

**SUPPLEMENT 2: Methodology**

**Details of Instruments used –**

| **Experiment** | **Instrument** | **Company** |
| --- | --- | --- |
| Prothrombin time | coagulation analyzer | Diagnostica Stago, France |
| Thrombin Time | coagulation analyzer | Diagnostica Stago, France |
| activated Partial Thromboplastin Time | coagulation analyzer | Diagnostica Stago, France |
| Fibrinogen | coagulation analyzer | Diagnostica Stago, France |
| Hemogram | Sysmex kx21 Hematology Analyzer | Sysmex Corporation, Japan |
| ELISA and activity assay | Synergy hybride H4 Multimode spectrophotometer | BioTek, VT, US |
| DNA quantitation | nanodrop 2000 Spectrophotometer | Thermo Fischer, USA |
| Thrombophilia | STA compact analyzer | Diagnostica Stago, France |

**Details of the various molecular markers evaluated in the study -**

| **Anticoagulants** | **Fibrinolytic Pathway** | **Inflammatory** | **Inflammatory-Procoagulant** | **Procoagulant** |
| --- | --- | --- | --- | --- |
| TAT | PAP | MPO | VCAM-1 | Fxa |
| TFPI | tPA | CRP | P-Selectin | TAFI |
| EPCR | uPA | VEGFR3 | ICAM-1 | FVIIa |
| TMBO |  |  | CD40L | VWF |
|  |  |  | MCP-1 |  |

***Molecular marker estimation:*** We evaluated the coagulative variables, fibrinolytic and platelet activation markers at each time point. Endothelial and inflammatory molecules were also analyzed. Anticoagulants like Tissue factor pathway inhibitor (TFPI), Tissue plasminogen activator (tPA), urokinase plasminogen activator (uPA), Endothelial protein C receptor (EPCR), and Thrombomodulin (TMBO) were assessed by sandwich ELISA (R&D Systems, MN, USA). The amount of Thrombin-antithrombin complex (TAT) and Plasmin-antiplasmin complex (PAP) amount in the plasma samples of subjects was measured along with platelet - endothelial activation and inflammatory response molecules like soluble P-selectin, CD40 ligand, Soluble C-reactive protein (sCRP), Myeloperoxidase (MPO) and Monocyte chemoattractant protein-1 (MCP-1), vascular cell adhesion molecule l (VCAM1), Intercellular adhesion molecule 1 (ICAM1), and Vascular endothelial growth factor receptor 3 (VEGFR3) using Duo-set ELISA kit from R&D Systems, MN, USA by following the manufacturer protocol. Furthermore, activity assays of procoagulants like Factor VIIa, Factor Xa, Thrombin activatable fibrinolysis inhibitor (TAFI), and Von Willebrand factor (vWF) were performed to assess the activation rate of these molecules by their respective activity kit from Sekisui diagnostics, CT, USA. Absorbance was taken using Synergy hybride H4 from biotek, VT, US, at 450 nm with blank correction at 540 nm. Concentration was presented in pg/mL or ng/mL for ELISA and in mU/mL for activity assays.

***Restriction fragment length polymorphism [RFLP] based Genotyping:*** For genetic analysis peripheral blood samples were collected in EDTA vacutainers [BD biosciences, CA, USA]. High molecular weight DNA was extracted from peripheral blood by QIAamp DNA isolation kit [Qiagen, Germany], using manufacturer’s protocol. Quantitative analysis of genomic DNA was done using DNA/RNA nanodrop 2000 spectrophotometer [Thermo Fischer, USA]. For qualitative analysis, samples [100ng/µl] were loaded on 0.7% agarose gel containing ethidium bromide, run for ~20min and visualized under UV. Desired gene sequences were amplified using specific PCR primers [as detailed in Table 1]. The final PCR reaction contained 100ng of DNA, 200μM deoxyribonucleotide triphosphate [dNTP], 10pmol of each primer and 0.6U taq polymerase in total volume of 25μl reaction buffer [50mM KCL, 20mM Tris-HCL, pH 8.3]. The amplified PCR products were digested with specific restriction enzymes at optimized temperature. Digested PCR products were mixed with DNA loading dye and subjected to agarose gel electrophoresis at varying concentrations ranging from 1.5 to 3.5 % based on the band sizes to be obtained after digestion SNP-positive [sample with known profile] and SNP-negative [blank] were used for verifying results in each experiment. Complete details is provided in the table below.

**Candidate genes SNPs primer sequences, PCR product size, annealing temperature, restriction enzyme and the fragment sizes**

| **Gene** | **Polymorphism,**  **Region** | **Primer Details** | **PCR Product (bp)** | **Annealing Temp** | **Restriction enzyme** | **Band Size (bp)** |
| --- | --- | --- | --- | --- | --- | --- |
| Factor V  Leiden | 1691 G/A,  rs6025 | F: TCAGGCAGGAACAACACCAT  R: GGTTACTTCAAGGACAAAATACCTGTAAAGCT | 241 | 58°C | *Hind III* | G=241  A=209, 32 |
| Prothrombin | 20210G/A, 3’UTRrs1799963 | F: ATTGATCAGTTTGGAGAGTAGGGG  R: AATAGCACTGGGAGCATTGAAGCT | 142 | 60°C | *Hind III* | G=142  A=119, 23 |
| TFPI | -536C/T, intron7 | F: TCTATTTTAATTGGCTGTAT  R: GCATGATAATAGTTTCCTGG | 170 | 65°C | *BseN1* | C=170  T=143, 27 |
| PAI-1 | 4G/5G, -675 promoter rs1799889 | F: CACAGAGAGAGTCTGGCCACGT  R: CCAACAGAGGACTCTTGGTCT | 98 | 60°C | *Bsl I* | 4G=98  5G=77, 22 |
| PAI-1 | -844G/A, 3’-UTR  rs2227631 | F: CAGGCTCCCACTGATTCTAC  R: GAGGGCTCTCTTGTGTCAAC | 510 | 60⁰C | *XhoI* | G=510  A=364, 146 |
| MTHFR | 677C/T, rs1801133 | F: TGAAGGAGAAGGTGTCTGCGGGA  R: AGGACGGTGCGGTGAGA | 198 | 62°C | *Hinf I* | C=198  T=175, 23 |
| MTHFR | 1298A/C, rs1801131 | F: CTTTGGGGAGCTGAAGGACTACTAC  F: CAATTTGTGACCATTCCGGTTTG | 163 | 62°C | *MboII* | A=56,31,30,28,18  C=84, 31, 30, 18 |
| Fibrinogen- β | 148C/T, promoter  rs1800787 | F: CCTAACTTCCCATCATTTTGTCCAATAAA  R: TGTCGTTGACACCTTGGGACTTAACTAG | 362 | 53°C | *Hind III* | C=265, 97  T=362 |
| β- Fibrinogen- β | -455G/A, promoter  rs18000790 | F: GCTTGTGGGAAATGAAGGAA  R: GGCAACCACTAAAATCGTGA | 469 | 59.5°C | *HaeIII* | A=469, 26  G=383, 86, 26 |
| Endothelial protein C receptor (EPCR) | 6936A/G Rs867186 | F.P. -GCTTCAGTCGTTGGTAAAC  R.P.-TCTGGCTTCACAGTGAGCTG | 314 | 57 | PstI | 314- GG  314, 195, 119- GA  119, 195- AA |
| Angiotensin converting enzyme (ACE) | I/D Rs1799752 | F.P. -CTGGAGACCACTCCCATCCTTTCT  R.P.-GATGTGGCCATCACATTCGTCAGAT | 490 | 65 | *-* | 490-II  490, 190- ID |
| Vascular endothelial growth factor (VEGF) | 936C/T Rs3025039 | F.P. -AAGGAAGAGGAGACTCTGCGCAGAGC  R.P.-AAATGTATGTATGTGGGTGGGTGTGTCTACAGG | 208 | 65 | *NlaIII* | 208- CC  208, 122, 86- CT  122, 86- TT |
| Endothelin-1 (EDN-1) | 8002G/A Rs2071942 | F.P. -CAAACCGATGTCCTCTGTA  R.P.-ACCAAACACATTTCCCTA | 358 | 52 | *TaqI* | 358-AA  358, 208, 150- AG  208, 150- GG |

**SUPPLEMENT 3: DETAILS OF THE THROMBOTIC EVENTS IN THE STUDY COHORT**

| **Thrombotic event** | **n** | **Incidence in %** | **Incidence per lakh person-years at >15000ft (Mean 99 days stay)** |
| --- | --- | --- | --- |
| Venous thrombosis | 12 | 1.6 | 5,926 |
| CVT | 08 | 1.07 | 3,951 |
| DVT & DVT+ PE | 05 | 0.67 | 2,469 |
| Arterial thrombosis | 03 | 0.4 | 1,482 |
| Stroke | 01 | 0.13 | 494 |
| PAOD | 02 | 0.27 | 988 |
| Total | 15 | 2.0 | 7,407 |

Legend: CVT – cerebral venous thrombosis; DVT – Deep Vein Thrombosis; PE – Pulmonary embolism; PAOD – Peripheral Arterial Occlusive disease; n – numbers, ft - feet

**Supplement 4: The details of the (A) Coagulogram (B) Hemogram and (C) Molecular markers**

| **Parameter** | **Screening** | **N** | | **Mean** | | **Median** | | **Std Dev** | | **Min** | | **Max** | | **p value** |
| --- | --- | --- | --- | --- | --- | --- | --- | --- | --- | --- | --- | --- | --- | --- |
|  |  | **Controls** | **Cases** | **Controls** | **Cases** | **Controls** | **Cases** | **Controls** | **Cases** | **Controls** | **Cases** | **Controls** | **Cases** |  |
| 1. **Coagulogram** | | | | | | | | | | | | | | |
| TT | Screening 1 | 35 | 8 | 17.39 | 16.26 | 16.70 | 16.65 | 2.60 | 2.47 | 13.50 | 11.70 | 26.10 | 19.80 | 0.5021 |
|  | Screening 2* | 35 | 8 | 16.01 | 16.04 | 15.90 | 16.50 | 1.56 | 1.41 | 11.30 | 13.50 | 19.30 | 18.00 | 0.9639 |
|  | Screening 3 | 35 | 0 | 19.17 | . | 18.00 | . | 4.66 | . | 13.90 | . | 39.30 | . |  |
| Fibrinogen | Screening 1* | 35 | 8 | 281.14 | 274.50 | 274.60 | 282.40 | 49.71 | 41.69 | 201.00 | 210.00 | 400.00 | 319.00 | 0.7023 |
|  | Screening 2 | 35 | 8 | 295.75 | 342.05 | 288.10 | 323.50 | 45.08 | 69.12 | 192.00 | 279.00 | 382.00 | 490.20 | 0.0679 |
|  | Screening 3* | 35 | 0 | 255.41 | . | 241.00 | . | 54.24 | . | 162.00 | . | 386.00 | . |  |

| **Parameter** | **Screening** | **N** | | **Mean** | | **Median** | | **Std Dev** | | **Min** | | **Max** | | **p value** |
| --- | --- | --- | --- | --- | --- | --- | --- | --- | --- | --- | --- | --- | --- | --- |
|  |  | **Controls** | **Cases** | **Controls** | **Cases** | **Controls** | **Cases** | **Controls** | **Cases** | **Controls** | **Cases** | **Controls** | **Cases** |  |
| 1. **Hemogram** | | | | | | | | | | | | | | |
| RBC | Screening 1* | 22 | 8 | 4.32 | 4.43 | 4.05 | 4.35 | 0.83 | 1.13 | 2.94 | 2.70 | 6.73 | 6.38 | 0.5735 |
|  | Screening 2* | 22 | 7 | 5.65 | 4.42 | 5.67 | 4.47 | 0.95 | 0.94 | 4.32 | 3.06 | 8.48 | 5.96 | **0.0134** |
|  | Screening 3* | 22 | 0 | 6.68 | . | 6.88 | . | 1.01 | . | 4.90 | . | 8.37 | . |  |
| MCH | Screening 1* | 22 | 8 | 29.55 | 28.63 | 29.40 | 29.10 | 2.97 | 2.97 | 22.30 | 22.70 | 35.90 | 32.40 | 0.4625 |
|  | Screening 2* | 22 | 7 | 38.14 | 38.26 | 37.75 | 34.20 | 6.01 | 8.68 | 22.70 | 28.80 | 47.60 | 52.60 | 0.9735 |
|  | Screening 3* | 22 | 0 | 27.94 | . | 27.65 | . | 3.97 | . | 21.30 | . | 36.50 | . |  |
| MCHC | Screening 1 | 22 | 8 | 33.41 | 32.76 | 33.75 | 33.15 | 2.73 | 1.94 | 23.30 | 28.80 | 36.60 | 34.70 | 0.2806 |
|  | Screening 2 | 22 | 7 | 48.25 | 46.46 | 46.40 | 44.90 | 8.89 | 9.56 | 25.40 | 30.60 | 60.80 | 59.40 | 0.231 |
|  | Screening 3* | 22 | 0 | 32.68 | . | 32.85 | . | 2.12 | . | 29.00 | . | 37.10 | . |  |
| MCV | Screening 1 | 22 | 8 | 88.72 | 89.46 | 87.30 | 87.75 | 6.85 | 5.90 | 81.20 | 83.80 | 110.50 | 97.60 | 0.5897 |
|  | Screening 2 | 22 | 7 | 72.69 | 83.11 | 70.79 | 80.00 | 7.28 | 8.09 | 65.43 | 75.60 | 93.40 | 94.10 | **0.0047** |
|  | Screening 3* | 22 | 0 | 85.08 | . | 84.65 | . | 6.96 | . | 72.20 | . | 98.90 | . |  |
| RDW | Screening 1* | 22 | 8 | 9.33 | 9.54 | 9.20 | 9.90 | 0.85 | 0.79 | 8.30 | 8.10 | 11.10 | 10.20 | 0.5394 |
|  | Screening 2 | 22 | 7 | 9.08 | 9.06 | 9.00 | 9.00 | 0.95 | 0.13 | 8.10 | 8.90 | 12.60 | 9.30 | 0.5175 |
|  | Screening 3* | 22 | 0 | 52.81 | . | 53.45 | . | 6.51 | . | 42.50 | . | 66.30 | . |  |
| MPV | Screening 1* | 22 | 8 | 9.15 | 9.04 | 9.25 | 9.15 | 0.50 | 0.72 | 8.40 | 7.90 | 10.50 | 10.00 | 0.6802 |
|  | Screening 2* | 22 | 7 | 7.60 | 7.51 | 7.65 | 7.50 | 1.02 | 1.05 | 5.80 | 6.30 | 9.60 | 9.60 | 0.8463 |
|  | Screening 3 | 22 | 0 | 9.20 | . | 9.65 | . | 2.24 | . | 0.00 | . | 11.10 | . |  |
| Pct | Screening 1 | 22 | 8 | 0.29 | 0.32 | 0.26 | 0.35 | 0.17 | 0.14 | 0.09 | 0.11 | 0.92 | 0.46 | 0.4668 |
|  | Screening 2* | 22 | 7 | 0.22 | 0.19 | 0.22 | 0.19 | 0.09 | 0.07 | 0.05 | 0.12 | 0.41 | 0.33 | 0.4884 |
|  | Screening 3 | 22 | 7 | 0.23 | 0.26 | 0.22 | 0.21 | 0.09 | 0.10 | 0.11 | 0.18 | 0.50 | 0.45 | 0.4748 |
| PDW | Screening 1 | 20 | 8 | 9.99 | 9.58 | 10.05 | 9.65 | 1.17 | 1.02 | 7.40 | 8.20 | 11.70 | 11.20 | 0.3707 |
|  | Screening 2* | 22 | 7 | 10.76 | 9.57 | 10.30 | 10.10 | 1.59 | 1.18 | 8.30 | 8.20 | 13.10 | 11.10 | 0.1196 |
|  | Screening 3 | 22 | 0 | 11.50 | . | 11.25 | . | 3.34 | . | 0.00 | . | 17.10 | . |  |

| **Parameter** | **Screening** | **N** | | **Mean** | | **Median** | | **Std Dev** | | **Min** | | **Max** | | **p value** |
| --- | --- | --- | --- | --- | --- | --- | --- | --- | --- | --- | --- | --- | --- | --- |
|  |  | **Controls** | **Cases** | **Controls** | **Cases** | **Controls** | **Cases** | **Controls** | **Cases** | **Controls** | **Cases** | **Controls** | **Cases** |  |
| 1. **Molecular Markers** | | | | | | | | | | | | | | |
| TFPI | Screening 1 | 41 | 8 | 16141.20 | 15236.75 | 16138.65 | 16043.00 | 298.18 | 3448.99 | 15624.70 | 10078.00 | 16836.33 | 19118.00 | 0.7764 |
|  | Screening 2 | 43 | 8 | 14923.38 | 11525.69 | 14969.19 | 13462.13 | 901.84 | 7444.56 | 13208.72 | 1506.71 | 16864.54 | 22130.00 | 0.2386 |
|  | Screening 3* | 43 | 13 | 12075.66 | 14772.69 | 12563.33 | 13958.00 | 3749.89 | 5223.96 | 2012.50 | 3678.00 | 19875.00 | 22458.00 | 0.1027 |
| P-Selectin | Screening 1 | 41 | 8 | 11417.19 | 12486.25 | 11289.23 | 12722.50 | 1206.34 | 9246.14 | 9350.77 | 3360.00 | 14243.08 | 31110.00 | 0.4735 |
|  | Screening 2 | 43 | 8 | 16295.22 | 34857.75 | 16189.92 | 30510.23 | 757.66 | 17756.12 | 14236.08 | 17452.15 | 18543.77 | 61500.00 | **<0.01** |
|  | Screening 3 | 43 | 13 | 26125.47 | 90989.91 | 24810.00 | 95717.82 | 10206.99 | 31218.30 | 9060.00 | 43960.40 | 51945.00 | 140841.58 | **<0.0001** |
| CRP | Screening 1 | 41 | 8 | 13571.39 | 14785.63 | 13146.34 | 14736.00 | 2299.35 | 2618.47 | 10634.15 | 11256.00 | 25170.73 | 18376.00 | 0.1383 |
|  | Screening 2 | 43 | 8 | 25753.94 | 110992.50 | 23707.32 | 106090.00 | 5078.79 | 75618.01 | 20170.73 | 19080.00 | 40378.05 | 203040.00 | **0.0017** |
|  | Screening 3* | 44 | 13 | 52754.89 | 96028.46 | 50080.00 | 96875.00 | 19463.84 | 18319.35 | 9880.00 | 65987.00 | 95480.00 | 133125.00 | **0.0010** |
| MPO | Screening 1 | 41 | 8 | 21803.24 | 33666.60 | 20272.75 | 29049.24 | 8594.72 | 18458.11 | 8522.75 | 17206.42 | 45022.80 | 77805.26 | **0.0315** |
|  | Screening 2 | 43 | 8 | 41726.74 | 240412.41 | 41777.50 | 147640.00 | 9078.11 | 178627.28 | 23340.00 | 59540.00 | 59152.55 | 491785.71 | **<0.0001** |
|  | Screening 3 | 44 | 13 | 28910.81 | 144600.71 | 32303.33 | 147963.20 | 13227.97 | 28010.87 | 3622.22 | 87903.60 | 68340.00 | 183015.79 | **<0.0001** |
| MCP-1 | Screening 1 | 23 | 8 | 123.46 | 188.75 | 95.00 | 170.00 | 114.51 | 82.71 | 15.00 | 110.00 | 425.00 | 360.00 | **0.0491** |
|  | Screening 2* | 31 | 8 | 401.60 | 313.33 | 375.50 | 331.67 | 131.53 | 88.34 | 120.00 | 190.00 | 722.00 | 448.33 | **0.0385** |
|  | Screening 3 | 40 | 13 | 683.48 | 977.60 | 602.50 | 566.92 | 325.43 | 1306.10 | 20.00 | 482.31 | 1750.00 | 5290.00 | 0.4756 |
| CD40L | Screening 1 | 41 | 8 | 717.07 | 1093.49 | 670.00 | 688.23 | 437.19 | 980.20 | 140.00 | 514.29 | 2020.00 | 3371.43 | 0.2183 |
|  | Screening 2 | 43 | 8 | 1010.11 | 21377.59 | 970.00 | 1649.29 | 379.90 | 27842.73 | 520.00 | 1444.29 | 2460.00 | 61500.00 | **<0.0001** |
|  | Screening 3 | 44 | 13 | 1576.45 | 2199.19 | 1282.50 | 2132.50 | 693.72 | 581.83 | 947.50 | 1235.50 | 3575.00 | 3237.50 | **0.0020** |
| EPCR | Screening 1* | 41 | 8 | 9097.12 | 9230.42 | 8790.00 | 9550.00 | 2509.14 | 2253.06 | 5240.00 | 6216.67 | 14840.00 | 12050.00 | 0.8834 |
|  | Screening 2 | 43 | 8 | 6803.83 | 8278.13 | 6470.00 | 8260.00 | 1325.34 | 1813.59 | 4770.00 | 6160.00 | 10570.00 | 11916.67 | **0.0191** |
|  | Screening 3 | 40 | 13 | 13463.92 | 6847.28 | 7660.00 | 6421.67 | 18298.22 | 2164.87 | 1310.00 | 3216.67 | 101041.67 | 10800.00 | 0.3364 |
| TMBO | Screening 1 | 41 | 8 | 2629.38 | 3075.06 | 2405.34 | 2485.80 | 911.82 | 1494.18 | 1491.06 | 1673.33 | 6233.91 | 5840.00 | 0.4903 |
|  | Screening 2 | 43 | 8 | 2210.42 | 1649.56 | 2216.29 | 1298.25 | 161.97 | 918.05 | 1902.00 | 668.75 | 2716.29 | 2981.25 | 0.0600 |
|  | Screening 3 | 44 | 13 | 2967.53 | 2478.75 | 2515.00 | 2440.00 | 1885.22 | 1192.22 | 420.00 | 717.78 | 8572.22 | 5806.67 | 0.5878 |
| tPA | Screening 1 | 41 | 8 | 2.27 | 3.46 | 2.28 | 2.23 | 1.07 | 2.73 | 0.70 | 1.14 | 5.40 | 9.67 | 0.3507 |
|  | Screening 2 | 43 | 8 | 2.76 | 3.31 | 2.51 | 3.16 | 1.05 | 0.88 | 1.50 | 2.12 | 6.39 | 4.82 | 0.6790 |
|  | Screening 3 | 44 | 13 | 2.95 | 3.31 | 2.71 | 2.39 | 1.06 | 3.19 | 1.76 | 1.53 | 6.72 | 13.76 | 0.4640 |
| TAT | Screening 1 | 41 | 8 | 5011.03 | 4472.36 | 3064.94 | 3939.42 | 6687.23 | 1475.76 | 305.65 | 2571.43 | 35395.02 | 7285.71 | 0.2977 |
|  | Screening 2 | 43 | 8 | 5439.52 | 8008.12 | 2932.86 | 8043.23 | 7456.56 | 1959.47 | 338.57 | 5000.00 | 35397.94 | 12000.00 | **0.0014** |
|  | Screening 3 | 43 | 13 | 5164.25 | 11084.88 | 3571.43 | 8500.00 | 6520.52 | 7374.03 | 428.57 | 3285.71 | 35285.71 | 27857.14 | **0.0002** |
| PAP | Screening 1 | 41 | 8 | 92.12 | 110.03 | 86.03 | 104.95 | 37.24 | 16.15 | 36.86 | 93.22 | 245.52 | 143.75 | **0.0499** |
|  | Screening 2 | 43 | 8 | 93.52 | 781.00 | 87.15 | 806.00 | 37.41 | 217.48 | 37.85 | 408.00 | 247.15 | 1176.00 | **<0.0001** |
|  | Screening 3 | 44 | 13 | 103.42 | 845.85 | 97.40 | 841.00 | 36.94 | 162.17 | 47.50 | 587.00 | 258.80 | 1140.00 | **<0.0001** |
| vWF | Screening 1 | 41 | 8 | 83.52 | 42.24 | 38.76 | 35.38 | 215.17 | 23.85 | 1.13 | 20.12 | 1400.58 | 98.84 | 0.8604 |
|  | Screening 2 | 43 | 8 | 56.50 | 74.12 | 43.74 | 70.64 | 44.25 | 31.70 | 6.11 | 24.35 | 158.82 | 122.33 | 0.1171 |
|  | Screening 3 | 44 | 13 | 58.21 | 73.68 | 45.59 | 78.50 | 44.64 | 34.91 | 8.13 | 20.32 | 166.14 | 125.39 | 0.1060 |
| TAFI | Screening 1 | 41 | 8 | 200.47 | 138.32 | 167.10 | 151.87 | 120.08 | 39.78 | 69.44 | 74.42 | 770.17 | 187.27 | 0.0909 |
|  | Screening 2 | 43 | 8 | 199.52 | 309.25 | 173.30 | 312.37 | 92.41 | 44.34 | 74.42 | 243.38 | 475.16 | 359.56 | **0.0015** |
|  | Screening 3 | 44 | 13 | 215.83 | 457.44 | 183.09 | 416.91 | 136.92 | 92.58 | 86.76 | 369.12 | 787.50 | 711.03 | **<0.0001** |
| Fxa | Screening 1 | 41 | 8 | 1883.60 | 800.74 | 526.90 | 641.82 | 2398.39 | 421.40 | 212.34 | 402.39 | 8918.22 | 1506.12 | 0.8181 |
|  | Screening 2 | 43 | 8 | 1866.64 | 4354.30 | 559.82 | 4028.85 | 2390.52 | 1609.60 | 245.27 | 2687.26 | 8951.14 | 7917.04 | **0.0028** |
|  | Screening 3 | 44 | 13 | 1871.96 | 8295.96 | 493.57 | 7893.70 | 2413.26 | 1760.37 | 290.16 | 6012.58 | 9041.14 | 12242.36 | **<0.0001** |
| FVIIa | Screening 1* | 41 | 8 | 25.65 | 24.26 | 25.07 | 24.03 | 7.90 | 4.65 | 10.45 | 18.64 | 45.56 | 30.57 | 0.5084 |
|  | Screening 2* | 43 | 8 | 27.34 | 30.21 | 26.92 | 30.73 | 8.28 | 4.91 | 9.47 | 23.42 | 46.67 | 38.35 | 0.2003 |
|  | Screening 3 | 39 | 13 | 29.15 | 51.91 | 27.43 | 45.42 | 8.00 | 20.95 | 12.81 | 40.32 | 45.42 | 118.54 | **<0.0001** |
| ICAM-1 | Screening 1 | 41 | 8 | 11386.15 | 13557.50 | 12312.00 | 13040.00 | 8282.27 | 2089.96 | 1032.00 | 11020.00 | 29070.00 | 17460.00 | 0.3793 |
|  | Screening 2 | 43 | 8 | 15744.99 | 26617.50 | 14070.00 | 24270.00 | 5318.92 | 5869.89 | 8110.00 | 21400.00 | 31990.00 | 39240.00 | **0.0002** |
|  | Screening 3 | 44 | 13 | 23864.55 | 47026.15 | 24060.00 | 48060.00 | 6987.36 | 6853.75 | 13060.00 | 36060.00 | 51460.00 | 56260.00 | **<0.0001** |
| VCAM-1 | Screening 1* | 41 | 7 | 1122.73 | 1073.97 | 1162.91 | 1012.73 | 157.23 | 220.09 | 740.91 | 830.91 | 1396.45 | 1432.50 | 0.5913 |
|  | Screening 2 | 43 | 8 | 6082.17 | 10812.65 | 5921.50 | 10841.70 | 650.05 | 974.03 | 5057.87 | 9418.47 | 8183.48 | 12032.56 | **<0.0001** |
|  | Screening 3 | 44 | 13 | 9895.79 | 20825.82 | 9894.55 | 20640.00 | 2164.96 | 2513.91 | 4603.64 | 17150.34 | 13603.64 | 24754.71 | **<0.0001** |
| uPA | Screening 1* | 41 | 8 | 46785.30 | 43288.13 | 46397.50 | 41372.50 | 4090.34 | 5278.88 | 39047.50 | 38222.50 | 58347.50 | 51722.50 | 0.1111 |
|  | Screening 2 | 43 | 8 | 4567.38 | 6006.88 | 4372.50 | 5260.00 | 1077.92 | 1988.45 | 2897.50 | 3797.50 | 9847.50 | 9847.50 | **0.0143** |
|  | Screening 3 | 44 | 13 | 1421.45 | 1057.08 | 1396.00 | 1026.00 | 105.77 | 110.84 | 1256.00 | 870.00 | 1816.00 | 1298.00 | **<0.0001** |
| VEGFR3 | Screening 1* | 41 | 8 | 29252.03 | 31750.00 | 27666.67 | 33833.34 | 8522.12 | 7088.44 | 15333.33 | 22000.00 | 54666.67 | 39333.33 | 0.3970 |
|  | Screening 2* | 43 | 8 | 45861.59 | 52750.00 | 45334.35 | 51000.00 | 7827.26 | 5983.44 | 30334.35 | 46666.67 | 69001.02 | 65666.66 | **0.0150** |
|  | Screening 3* | 44 | 13 | 46864.09 | 64187.69 | 47430.00 | 63480.00 | 7610.46 | 4342.90 | 31980.00 | 58880.00 | 62580.00 | 75480.00 | **<0.0001** |

***Acronyms used: (N – number in each group; Std Dev – Standard Deviation; Min – Minimum, Max – Maximum; TT – thrombin time (s); Fibrinogen (mg/dl); RBC – Red blood cell count (million/µL); MCH – Mean corpuscular hemoglobin (g/dl); MCHC – mean corpuscular hemoglobin concentration; MCV – Mean corpuscular volume (fL); RDW – Red cell distribution width; MPV – mean platelet volume (fL); Pct – platelet crit (%); PDW – Platelet distribution width); TFPI - Tissue factor pathway inhibitor (pg/ml); P-Selectin (pg/ml); CRP - C reactive protein (pg/ml); MPO – Myeloperoxidase (pg/ml); MCP-1 - Monocyte chemoattractant protein-1 (pg/ml); CD40L - CD_40_ ligand (pg/ml); EPCR - Endothelial protein C receptor (pg/ml); TMBO - thrombomodulin (pg/ml); tPA - tissue plasminogen activator (pg/ml); TAT - Thrombin antithrombin complexes (pg/ml); PAP - Plasmin antiplasmin complexes (pg/ml); vWF - Von Willebrand factor (mU/ml); TAFI - Thrombin activated fibrinolysis inhibitor (pg/ml); FXa - Factor X activated (mU/ml); FVIIa - Factor VII activated (mU/ml); ICAM-1 - Intercellular Adhesion Molecule 1 (pg/ml); VCAM-1 - Vascular cell adhesion molecule type 1 (pg/ml); uPA - Urokinase type plasminogen activator (pg/ml); VEGFR3 - Vascular endothelial growth factor receptor 3 (pg/ml)***

***Legend: Asterisk (*) on phases indicate normal distribution of the parameter, lack of asterisk indicates not normal distribution; Bold in p values indicate statistically significant; Underlined p values indicate analysis by Wilcoxon test, those which are not underlined were analyzed by student’s t test. Missing data was addressed by deletion of rows for time-based/repeated measures analysis***

*.*

| Supplement 5. Contingency test for thrombosis associated SNPs between patients and study control subjects | | | | | | |
| --- | --- | --- | --- | --- | --- | --- |
| Gene | Polymorphism, region | χ^2^ | p-value | Fisher exact | OR | Change (95%) |
| Factor V Leiden (FVL) | 1691 G/A rs6025 | *Monomorphic (no mutation observed)* | | | | |
| Prothrombin | 20210 G/A, 3’”UTR rs1799963 | *Monomorphic (no mutation observed)* | | | | |
| Tissue factor pathway inhibitor (TFPI) | -536 C/T, intron7 | *Monomorphic (no mutation observed)* | | | | |
| Plasminogen activator inhibitor type- 1 (PAI-1) | 4G/5G, -675 promoter rs1799889 | 25.43 | <0.0001 | 0.0041 | 0.046 | 0.0025 – 0.84 |
| Plasminogen activator inhibitor type- 1 (PAI-1) | -844 G/A, 3ÜTR rs2227631 | 5.70 | 0.0578 | *Only Heterozygous* | | |
| Methylene tetrahydrofolate reductase (MTHFR) | 677 C/T rs1801133 | 26.75 | <0.0001 | 0.0003 | 155.4 | 6.187 - 3903 |
| Methylene tetrahydrofolate reductase (MTHFR) | 1298 A/C, rs1801131 | *no mutation observed* | | | | |
| Fibrinogen-β | 148C/T, promoter rs1800787 | 0.715 | 0.699 | 1 | 0.6364 | 0.031 – 12.72 |
| Fibrinogen-β | -455 G/A, promoter rs18000790 | 4.294 | 0.116 | 1 | 0.733 | 0.037 – 14.35 |
| Endothelial protein C receptor (EPCR) | 6936A/G rs867186 | *Monomorphic (no mutation observed)* | | | | |
| Angiotensin converting enzyme (ACE) | I/D rs1799752 | 0.92 | 0.63 | 0.65 | 0.346 | 0.037 – 3.255 |
| Vascular endothelial growth factor (VEGF) | 936C/T rs3025039 | *Monomorphic (no mutation observed)* | | | | |
| Endothelin-1 (EDN- 1) | 8002G/A rs2071942 | 2.407 | 0.30 | *Only Heterozygous* | | |

**** were assigned for High Altitude Studies

Continued----

| Supplement 5. Contingency test for thrombosis associated SNPs between patients and study control subjects | | | | | | |
| --- | --- | --- | --- | --- | --- | --- |
| Gene | Polymorphism, region | χ^2^ | p-value | Fisher exact | OR | Change (95%) |
| Factor V Leiden (FVL) | 1691 G/A rs6025 | *Monomorphic (no mutation observed)* | | | | |
| Prothrombin | 20210 G/A, 3’”UTR rs1799963 | *Monomorphic (no mutation observed)* | | | | |
| Tissue factor pathway inhibitor (TFPI) | -536 C/T, intron7 | *Monomorphic (no mutation observed)* | | | | |
| Plasminogen activator inhibitor type- 1 (PAI-1) | 4G/5G, -675 promoter rs1799889 | 25.43 | <0.0001 | 0.0041 | 0.046 | 0.0025 – 0.84 |
| Plasminogen activator inhibitor type- 1 (PAI-1) | -844 G/A, 3ÜTR rs2227631 | 5.70 | 0.0578 | *Only Heterozygous* | | |
| Methylene tetrahydrofolate reductase (MTHFR) | 677 C/T rs1801133 | 26.75 | <0.0001 | 0.0003 | 155.4 | 6.187 - 3903 |
| Methylene tetrahydrofolate reductase (MTHFR) | 1298 A/C, rs1801131 | *no mutation observed* | | | | |
| Fibrinogen-β | 148C/T, promoter rs1800787 | 0.715 | 0.699 | 1 | 0.6364 | 0.031 – 12.72 |
| Fibrinogen-β | -455 G/A, promoter rs18000790 | 4.294 | 0.116 | 1 | 0.733 | 0.037 – 14.35 |
| Endothelial protein C receptor (EPCR) | 6936A/G rs867186 | *Monomorphic (no mutation observed)* | | | | |
| Angiotensin converting enzyme (ACE) | I/D rs1799752 | 0.92 | 0.63 | 0.65 | 0.346 | 0.037 – 3.255 |
| Vascular endothelial growth factor (VEGF) | 936C/T rs3025039 | *Monomorphic (no mutation observed)* | | | | |
| Endothelin-1 (EDN- 1) | 8002G/A rs2071942 | 2.407 | 0.30 | *Only Heterozygous* | | |

**** were assigned for High Altitude Studies
